# Reciprocal relationships between sleep and smell

**DOI:** 10.3389/fncir.2022.1076354

**Published:** 2022-12-22

**Authors:** Giuliano Gaeta, Donald A. Wilson

**Affiliations:** ^1^Givaudan UK Limited, Health and Well-Being Centre of Excellence, Ashford, United Kingdom; ^2^Emotional Brain Institute, Nathan Kline Institute for Psychiatric Research, Orangeburg, NY, United States; ^3^Child and Adolescent Psychiatry, NYU School of Medicine, New York University, New York, NY, United States

**Keywords:** olfaction, odor perception, sleep, insomnia, fragrance, memory

## Abstract

Despite major anatomical differences with other mammalian sensory systems, olfaction shares with those systems a modulation by sleep/wake states. Sleep modulates odor sensitivity and serves as an important regulator of both perceptual and associative odor memory. In addition, however, olfaction also has an important modulatory impact on sleep. Odors can affect the latency to sleep onset, as well as the quality and duration of sleep. Olfactory modulation of sleep may be mediated by direct synaptic interaction between the olfactory system and sleep control nuclei, and/or indirectly through odor modulation of arousal and respiration. This reciprocal interaction between sleep and olfaction presents novel opportunities for sleep related modulation of memory and perception, as well as development of non-pharmacological olfactory treatments of simple sleep disorders.

*Heraclitus: “Even a soul submerged in sleep is hard at work, and helps make something of the world.”*

## 1 Introduction

There is now a consensus that essentially all animals express some form of sleep, which is most generally defined as a reduction in movement and muscle activity, and reduced behavioral responsiveness to sensory input compared to wakefulness. In humans and other mammals, sleep is further characterized by distinct neurophysiological features and can be divided into different stages based on those features, including rapid eye movement (REM) sleep and slow-wave non-REM (nREM) sleep. While conscious and behavioral responsiveness to sensory stimuli is reduced during sleep, sensory systems can still respond to inputs during sleep in ways that may not induce arousal or disrupt the sleep cycle. For example, sensory stimulation during sleep can modulate sleep-dependent memory consolidation for items associated with the incoming sensory stimuli. In fact, in contrast to disrupting sleep, under some circumstances specific stimuli can enhance sleep quality, for example, by enhancing the amplitude of cortical slow-waves during slow-wave and non-REM sleep. Thus, far from being a period where the brain is off-line, sensory systems in the sleeping brain can continue to monitor the external environment at a non-arousing, sub-conscious level which can both modulate ongoing-sleep dependent processes and sleep itself. This suggests a reciprocal relationship between sleep and sensation, where sleep state can modulate sensory responsiveness and sensory input can modulate sleep.

In this review, we summarize this reciprocal interplay between sleep and sensation with a specific focus on olfaction, reviewing both how sleep shapes odor perception and memory and how odors can influence sleep quality. Olfaction is highlighted here given the dramatic differences in the anatomy of the olfactory system and all other mammalian sensory systems. However, we will also compare and contrast the olfaction-sleep relationship to that observed in other sensory systems where data exist.

## 2 Sleep

Broadly speaking, sleep provides the brain with an opportunity to adjust to and evaluate activity from the previous waking period, restore conditions necessary for optimal performance during the next waking period, and more efficiently remove metabolic wastes that could otherwise hamper function or even induce pathology. For example, neural activity during the waking period can enhance the strength of synapses between co-active neurons–a common form of information storage. However, not all of this synaptic strengthening conveys useful information and continued, unchecked strengthening could reduce the storage capacity of circuits as synapses become saturated. Sleep serves two functions that address these issues. First, synapses and circuits modified by memory storage of biologically meaningful or significant events can undergo sleep-dependent consolidation, allowing strengthening or transfer of those engrams for long-term storage (Stickgold, 2005; Dudai et al., 2015). In contrast, synaptic changes not related to such events can be re-set through sleep-dependent synaptic homeostasis, bringing those synapses back within the middle of their dynamic range and ready to respond to the events of the next waking period (Tononi and Cirelli, 2014). Sleep can also provide an opportunity to flush metabolic wastes from the brain. During sleep the glymphatic system which promotes flow of cerebrospinal fluid in the interstitial space, increases its bulk flow which allows greater removal of metabolic wastes such as small peptides (Xie et al., 2013). For example, during waking, levels of the Alzheimer’s disease pathogen amyloid-beta increase and then during slow-wave sleep they decrease through this sleep-dependent wash (Kang et al., 2009). Impaired sleep leads to a build-up of amyloid-beta (Shokri-Kojori et al., 2018). As a side note, elevated amyloid-beta in the brain leads to impaired sleep (Tabuchi et al., 2015), creating a positive feedback loop which may contribute to Alzheimer’s disease progression (Ju et al., 2014).

Sleep is not a unitary state but rather can be divided into multiple stages based on cortical and motor activity in healthy adults (Pace-Schott and Hobson, 2002). The different vigilance/sleep states contribute differently to sleep-dependent functions and can differently modulate sensitivity to sensory input. In humans, non-REM (nREM) sleep can be divided into four stages (Genzel et al., 2014; Leger et al., 2018). Over the course of a night the brain initially shifts from waking to Stage 1 sleep. Stage 1 is an intermediate stage between waking and light sleep with cortical activity dominated by theta band oscillations. In Stage 2, cortical sleep spindles and K-complexes emerge (see below). Stages 3 and 4 are characterized by large amplitude delta band cortical oscillations and are the stages of deepest sleep. The final sleep stage is rapid eye movement (REM) sleep which is characterized by lateral, oscillatory, eye movements, and a strong decrease in muscle tone elsewhere in the body. Cortical activity during REM sleep is very similar to that observed during waking with both states showing low amplitude, high frequency oscillations. Over the course of a typical night’s sleep in humans, the brain cycles between the slow-wave and REM states 5–6 times before waking in the morning. In rodents, sleep is largely limited to just REM and nREM (large amplitude delta oscillations) stages, though they may express a form of light sleep similar to human stages 1 and 2 (Genzel et al., 2014). Rodent sleep occurs in relatively short bouts (e.g., 10 min) over a 24 h period with higher probability during the light phase of the light-dark cycle.

The drive to initiate sleep is hypothesized to be mediated by two forces, sleep pressure induced by a period of waking (process S) and the circadian rhythm (process C) which modulates sleep based on time of day (Borbely et al., 2016). Sleep pressure not only increases the probability of sleep onset, it can modulate sleep physiology once initiated. For example, following a prolonged period of waking (i.e., high sleep pressure), the amplitude of delta band cortical slow-waves during nREM sleep is enhanced compared to sleep induced by lower sleep pressure.

**FIGURE 1:**
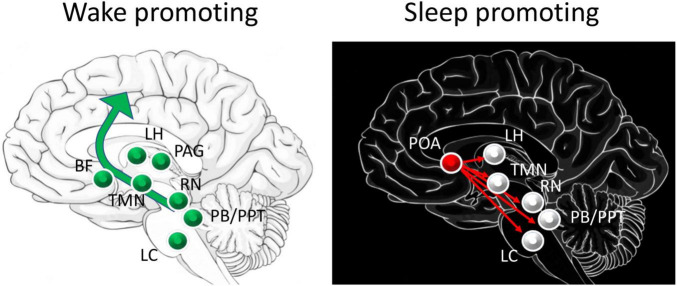
Schematic of major circuits involved in sleep and waking. Major direct contributors to arousal and waking are the excitatory projections from the pedunculopontine tegmental nucleus (PPT) and the parabrachial nucleus (PB) to basal forebrain (BF) that projects broadly throughout the cortex, including the olfactory cortex. Neurons in the lateral hypothalamus (LH) contribute to inhibition of sleep promoting regions. Dopamine release by the periaqueductal grey (PAG) area directly contributes to waking, while histamine from the tuberomammillary nucleus (TMN), serotonin from the raphe nucleus (RN) and norepinephrine from the locus coeruleus (LC) have more modulatory roles in promoting cortical arousal. Sleep promoting regions include the preoptic area (POA), specifically the ventrolateral preoptic (VLPO) and median preoptic (MnPO) areas which send GABAergic projections to major components of the waking system.

The transitions between wake and sleep and between sleep states, as well as state maintenance, are controlled by many regions including basal forebrain, hypothalamic, brainstem and thalamocortical circuits (Figure 1; Pace-Schott and Hobson, 2002; Saper et al., 2010; Lee and Dan, 2012; Lazarus et al., 2013; Gent et al., 2018; Ren et al., 2018; Redinbaugh et al., 2020). A short review of these sleep related circuits will provide an opportunity to identify sites of potential direct or indirect convergence with olfactory inputs that could underlie odor modulation of sleep discussed below.

Put very simply, these widely distributed brain regions can be divided into (1) wake promoting circuits, (2) sleep promoting circuits with distinct contributors to REM or nREM sleep, and (3) regions that function as switches to flip between these distinct physiological states (Pace-Schott and Hobson, 2002; Saper et al., 2010). Wake promoting circuits are most active during waking and less so during sleep, and in many cases have been shown to induce arousal when directly stimulated. These areas include ascending cholinergic neurons from the brainstem (e.g., pedunculopontine tegmental nuclei) that project to the thalamus, as well as basal forebrain cholinergic neurons that project widely across the cortex. Monoaminergic neurons in the raphe nucleus (serotonergic) and nucleus locus coeruleus (noradrenergic) also project broadly to hypothalamus, basal forebrain, and cortex. The lateral hypothalamic orexin neurons directly excite the cortex and basal forebrain as well as target many sources of the brainstem wake pathways. More recently it has been demonstrated that activity in the central lateral thalamus and its cortical targets correlates with waking and sleep, and selective stimulation of the central lateral thalamus can evoke arousal and waking (Redinbaugh et al., 2020). These diverse ascending pathways lead to cortical arousal and desynchronized, high frequency EEG oscillations characteristic of waking state.

Sleep promoting circuits function in part through suppression of the waking circuits. The preoptic area, especially the ventrolateral (VLPO) and median (MnPO) preoptic nuclei inhibit activity in brainstem and basal forebrain waking circuits (Pace-Schott and Hobson, 2002; Saper et al., 2010). These preoptic area nuclei are also targeted by the waking circuit in a mutually inhibitory manner such that the preoptic area can function as a switch to reliably shift between sleep and wake states (Saper et al., 2010). It should also be noted that these basal forebrain nuclei can include heterogeneous populations of neurons which each contribute uniquely to the sleep-wake cycle. For example, selective optogenetic activation of basal forebrain/pre-optic area inhibitory parvalbumin + neurons can promote waking, while activation of inhibitory somatostatin + neurons in the same region can promote sleep (Xu et al., 2015).

During sleep, the transitions between REM and nREM sleep appear to be controlled by reciprocally inhibitory circuits in the pons (Saper et al., 2010). This switch is modulated by monoamines and orexins in a manner similar to the sleep-wake switch. For example, monoaminergic neurons in the serotonergic raphe nucleus and noradrenergic locus coeruleus project to the pons where they suppress REM sleep by exciting REM-off neurons. During REM sleep the locus coeruleus is nearly silent, allowing REM-on neurons to dominate. Hypothalamic orexin neurons can also excite REM-off neurons. In contrast, brainstem cholinergic neurons can excite REM-on neurons and flip the system from nREM to REM sleep (Saper et al., 2010).

The main contributors to specific electrographic features of sleep have also been identified. For example, the slow, delta band oscillations characteristic of slow-wave and nREM sleep derive from a thalamocortical loop. During nREM sleep, cortical neurons display alternation between up-states, where firing rate can be as active as during waking, and down-states where firing rate is strongly suppressed (Steriade et al., 2001; Destexhe et al., 2007). This alternation is expressed in the EEG as a slow, 1–4 Hz, large amplitude, oscillation. Delta waves are generated by activity within a thalamocortical loop, with cortical projecting thalamic neurons displaying spontaneous bursting at delta frequency, and cortical neurons oscillating (up-state/down-state) in phase (Steriade, 1997; Buzsaki, 2006). Sleep stage 2 cortical sleep spindles are brief (1–3 s) 10–15 Hz oscillations which occur during slow-wave sleep, have been linked to memory consolidation, and are believed to disrupt sensory thalamic input to the cortex. Sleep spindles appear to be driven by the thalamic reticular nucleus, a GABAergic group of neurons projecting broadly to other thalamic nuclei and receiving a strong excitatory input from the neocortex (Luthi, 2014). K-complexes occur during Stage 2 sleep and are spontaneous or sensory evoked large positive-negative EEG potentials about 500 ms in duration, sometimes associated with sleep spindles (Colrain, 2005; Halasz, 2016). K-complexes are cortically generated and appear act as a synchronous corticothalamic input which helps synchronize other sleep related rhythms such as spindles and slow-waves (Amzica and Steriade, 2002). They are typically not localized to the specific sensory cortex tied to the initiating stimulus modality, but rather are generated in frontal regions.

This description of sleep and its mechanisms is not meant to be exhaustive, but rather to provide an anatomical and physiological framework upon which to place sleep-related sensory processing and perception, and specifically olfactory processing and odor modulation of sleep in the subsequent sections.

## 3 Sleep and sensory processing across systems

We begin our review of sleep and sensory processing with a brief overview of canonical thalamocortical systems that have received the most research attention in this area. This is not meant as an exhaustive review but rather allows later comparison with the olfactory system.

### 3.1 Thalamocortical systems

All mammalian sensory systems except olfaction share a basic circuit structure that enables detection, discrimination, and recognition (memory) of changes in the external, and in some cases internal, environment. The canonical circuit involves peripheral receptors (e.g., photoreceptors, auditory hair cells, corpuscular receptors located in the skin) innervated by neurons which project directly to the central nervous system–either to the spinal cord or directly to the brain where they target second order neurons. These second order neurons then directly or indirectly target thalamic nuclei that are relatively devoted to a single sensory pathway, and those thalamic neurons then project to the primary sensory neocortical region that is dominated by that sensory system. It should be noted that all sensory cortices appear to have some multi-modal sensitivity (Ghazanfar and Schroeder, 2006; Stein and Stanford, 2008). Conscious perception of activity in these sensory streams appears to require activity beyond the primary sensory regions in higher order sensory cortices (Blake and Logothetis, 2002; Koch et al., 2016), and perhaps prefrontal cortex (Kapoor et al., 2022), though there is continued debate over the neuroanatomy of conscious perception.

Conscious awareness of environmental and interceptive stimuli is reduced during sleep, and historically, the thalamus was seen as the “sensory gate” reducing information flow to the cortex during sleep. During rodent nREM sleep and human Stage 2 slow wave sleep, thalamic neurons that normally provide sensory input to the neocortex become hyperpolarized and shift to a bursting mode of firing, inducing “sleep spindles” in the cortical EEG (Luthi, 2014). This mode of firing is believed to be non-optimal for sensory transmission resulting therefore in sensory gating. However, response to sensory stimulation, while reduced during nREM sleep, can be observed within thalamocortical sensory pathways all the way to at least the primary sensory cortex (Livingstone and Hubel, 1981; Sela et al., 2016), suggesting at least some maintained sensory throughput. During Stage 2 slow-wave sleep cortical recordings show spontaneous and sensory evoked waveforms called K-complexes (Davis et al., 1939; Amzica and Steriade, 1997). The sensory-evoked K-complexes could reflect a sensory-evoked disturbance in sleep, making waking more likely. Alternatively, they have been hypothesized to reflect an effort to maintain sleep despite cortical sensory input (Amzica and Steriade, 2002; Halasz, 2016). Given sufficient sensory input (e.g., amplitude, modality), however, sleep will be disrupted and a state change to waking will be evoked. REM sleep is also protected from sensory evoked arousal, though Stage 4 nREM sleep appears to show the most extreme sensory gating (Pare and Llinas, 1995). In fact, impairments in sensory gating may contribute to insomnia. Poor auditory sensory gating has been observed in individuals with primary insomnia (i.e., impaired ability to initiate and maintain restful sleep) (Hairston et al., 2010). This has led to the hypothesis that insomnia derives in part from an impairment in the ability to filter sensory input (Bonnet and Arand, 1997; Hairston et al., 2010).

In thalamocortical systems, sensory evoked arousal from sleep most commonly involves activating regions within the widely distributed wake-promoting system described above (Jones, 2003; Calderon et al., 2016). Regions contributing to waking include neurons from diverse brain stem, hypothalamic, thalamic and basal forebrain regions that can project broadly to the cortex and evoke high frequency beta and gamma band oscillations and behavioral arousal. One set of targets of many of these regions are dorsal thalamic nuclei that in turn project broadly throughout the neocortex. Activation of these thalamic nuclei can directly induce cortical arousal (e.g., low amplitude, high frequency oscillations). Importantly, these distributed arousal inducing regions receive multisensory inputs, which could enhance or, potentially reduce, arousal and modulate the likelihood of sleep onset and/or maintenance.

While this discussion emphasizes the impact of sensory input on disrupting sleep and the processes within thalamocortical systems to reduce those impacts, sleep and sensation can have more complex relationships. For example, sleep and sensation can reciprocally interact. A potentially familiar example of a sensory system with a bi-directional relationship to sleep is nociception, which is consciously perceived as pain. Sleep deprivation can reduce the stimulus threshold for perceiving pain and increase the intensity of perceived pain (Tang et al., 2012; Krause et al., 2019). Thus, the duration and/or quality of prior sleep can affect the subsequent perception of pain. However, pain can also affect sleep. Pre-sleep pain can impair sleep onset and quality, though may do so by increasing general arousal rather than as a direct sensory modulation of sleep circuits (Riemann et al., 2010; Tang et al., 2012). As discussed in Section “Sleep and memory across systems,” there is also evidence for sensory stimulation and context to enhance sleep, either by reducing latency to sleep or improving sleep quality.

### 3.2 Olfactory system

**FIGURE 2:**
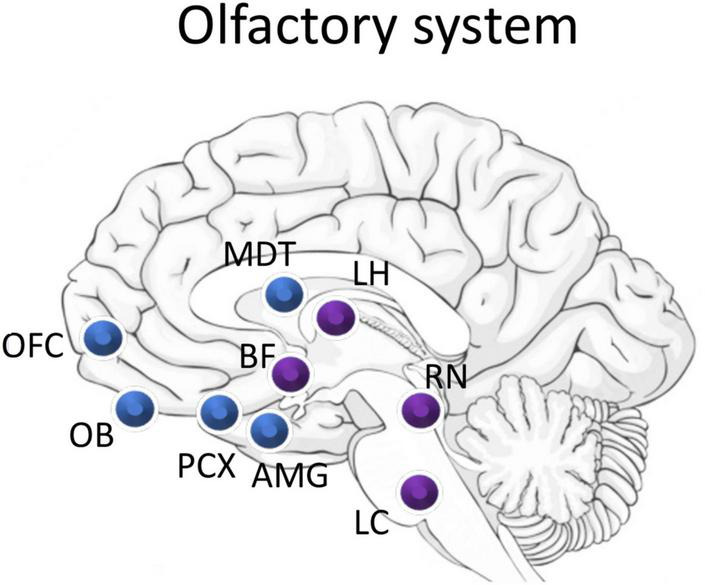
Schematic of major olfactory system components (blue) and overlap with sleep related areas (purple). Odor information flows from the olfactory bulb (OB) to the piriform cortex (PCX). PCX direct projection targets include the mediodorsal nucleus of the thalamus (MDT), the orbitofrontal cortex (OFC), the amygdala (AMG), and regions of the lateral hypothalamus (LH). The olfactory system receives strong neuromodulatory input from the cholinergic basal forebrain (BF), serotonergic raphe nucleus (RN), and the noradrenergic locus coeruleus (LC). Importantly, neurons in the LH, BF, and LC have all been shown to also be odor responsive.

As described above, the vast majority of work on the interplay between sensory processing and sleep has focused on thalamocortical sensory systems (e.g., vision, audition, touch). These systems share a similar anatomical structure, and, where tested, show similar sleep-wake state-dependence and modulation. The olfactory system in contrast, has a unique neuroanatomical pathway and may have a unique relationship with sleep (Figure 2; Shanahan and Gottfried, 2014; Perl et al., 2017).

The mammalian olfactory system begins with olfactory sensory neurons located in the olfactory epithelium of the nose. These sensory neurons express olfactory receptor proteins on their dendrites and send an axon directly into the forebrain to the olfactory bulb. The olfactory bulb is a cortical, laminated structure comprised of output neurons (mitral and tufted cells) which receive input from the sensory neurons and in turn project to a wide array of cortical areas including the anterior olfactory cortex, the olfactory tubercle (a.k.a. tubular striatum), the anterior and posterior piriform cortex, the cortical nucleus of the amygdala, and the lateral entorhinal cortex (Shipley and Ennis, 1996; Cleland and Linster, 2019). These regions that receive direct input from the olfactory bulb are known collectively as the olfactory cortex. The olfactory bulb also includes a very large number of interneurons (ratio of interneurons to projection neurons 50:1) which have a number of functions including lateral inhibition and controlling excitability of output neurons, as well as serving as the primary target of top-down inputs from most of the cortical targets just listed.

Although there is no thalamic nucleus between the periphery and olfactory cortex, it has been hypothesized that the olfactory bulb may serve some thalamic functions (Kay and Sherman, 2007). For example, similar to thalamic sensory nuclei, the olfactory bulb receives extensive neuromodulatory inputs, olfactory bulb output neurons show state-dependent shifts between tonic and burst firing modes, and through the targeting of inhibitory granule cells by top-down input, the bulb-cortex network shares important similarities with thalamocortical networks of the other senses (Kay and Sherman, 2007). In fact, local field potential oscillations recorded in the olfactory bulb can be used to classify vigilance states, and when combined with hippocampal theta can distinguish between REM and nREM sleep without requiring EMG (Bagur et al., 2018).

The olfactory cortex, containing neurons just two synapses from the nose, in turn projects broadly to regions including nuclei in the hypothalamus and thalamus, the orbitofrontal cortex, ventral striatum, hippocampus, and amygdala–all of which respond robustly to odors (Courtiol and Wilson, 2017; Cleland and Linster, 2019). The olfactory pathway does include a thalamic nucleus, the mediodorsal nucleus, which is a cortico-cortical pathway connecting, among other areas, the piriform cortex with the orbitofrontal cortex, and thus serves a much different role than that of thalamic nuclei in other sensory systems (Courtiol and Wilson, 2015). The olfactory cortex also receives extensive inputs from most of these regions as well as diverse multisensory inputs [e.g., taste (Maier et al., 2012), audition (Wesson and Wilson, 2010), vision (Gottfried and Dolan, 2003)], and extensive neuromodulatory inputs from the locus coeruleus, raphe nucleus, cholinergic basal forebrain regions, ventral tegmental dopaminergic neurons, and hypothalamic orexin neurons (Linster and Cleland, 2016; Julliard et al., 2017).

Thus, while there are major anatomical differences between the olfactory pathway and other sensory systems, key features important for the interplay between sensory processing and sleep exist. In fact, response to odors, both at the behavioral and electrophysiological levels is state-dependent, with clear evidence of sleep-dependent sensory gating and/or changes in functional connectivity in many cases (Shanahan and Kahnt, 2022). For example, in anesthetized rats undergoing spontaneous shifts between large amplitude slow-wave states and low amplitude fast-wave states, odor-evoked activity within rodent piriform cortex is greatly reduced during slow-wave activity (Murakami et al., 2005; Fontanini and Katz, 2008; Wilson, 2010). A similar decrease in piriform cortical odor-evoked activity during nREM sleep has been observed in unanesthetized rats (Barnes et al., 2011).

At the behavioral level sleep greatly reduces odor-evoked responses. In a small (*n* = 6) study using peppermint (which can also activate the trigeminal system) and pyridine odors, as well as 800 Hz tones, subjects were asked to push a button when they detected a stimulus. Stimuli were delivered during nREM stages I, II, and IV, as well as REM sleep. While tones evoked behavioral responses on 75% of trials regardless of sleep stage, odor rarely evoked responses in stage II, IV, or REM (Carskadon and Herz, 2004). In a similar study, Badia et al. (1990) tested peppermint odor-evoked EEG and autonomic responses in human subjects in stage II sleep. The subjects showed more arousals (e.g., HR, EEG) to odor than to clean air during sleep, though odor responses were greatly diminished during stage II bouts that were later during the night compared to those early in the night. In 3 day old human infants, the threshold for startle or respiratory responses to odor (isoamyl acetate) was significantly enhanced (i.e., required higher intensities) during slow-wave sleep but not affected during REM sleep (Murray and Campbell, 1970). Interestingly, in contrast to all other senses tested, odors do not evoke cortical K-complex responses during sleep in humans (Perl et al., 2016), which could either reflect the poor efficacy of odors to evoke arousals during sleep, or the unique architecture of the olfactory pathway. It should be noted that in addition to sleep effects on odor responsivity, circadian rhythms can also affect odor sensitivity in both humans (Herz et al., 2017) and rodents (Granados-Fuentes et al., 2006), with lowest threshold/peak responsiveness during the early night for humans and during the middle of the dark, active phase for rodents.

Together these studies support a sleep-dependent, sensory gate in olfaction, though with some variability between studies. In fact, optogenetic stimulation of olfactory sensory neurons during different vigilance states in mice suggests a lack of state-dependent gating as far as the anterior piriform cortex and orbitofrontal cortex—the latter of which receives both direct and indirect input from the anterior piriform cortex (Schreck et al., 2022). Schreck et al. (2022), suggesting that the reduced odor evoked activity during sleep seen in other paradigms may not be due to central sensory gating, but rather related to changes in respiration during sleep, which could reduce stimulus access to the receptors.

Thus, while response to odor is reduced during sleep, and especially nREM sleep, odor processing appears to occur across all vigilance states. One factor that may influence the extent of apparent sleep-dependent sensory gating is which measure of the odor response is used. For example, in contrast to the limited ability of odors to induce waking compared to auditory cues described above (Carskadon and Herz, 2004), the latency to wake from nREM sleep in response to threatening stimuli in mice is similar in a direct comparison of auditory, vestibular/somatosensory and olfactory modalities, where the odor was a predator odor (Iwakawa et al., 2018). This could suggest that the diversity of direct targets of olfactory bulb projection neurons may create parallel processing streams for olfactory information, some of which are state-dependent and some of which are not. For example, in rodents, piriform cortex–mediodorsal thalamic nucleus functional connectivity is reduced during slow-wave state compared to waking (Courtiol and Wilson, 2014) which may alter this indirect information flow between the piriform cortex and orbitofrontal cortex, while leaving direct piriform cortical—orbitofrontal cortex connectivity intact. How or whether sleep differentially affects olfactory bulb output to the amygdala, olfactory tubercle, posterior piriform cortex, or lateral entorhinal cortex is currently not known but could provide major insight into pathway or information specific, state-dependent effects on odor processing. Thus, for example, olfactory input could be very effective at driving some structures/pathways during sleep, but less effective at driving other regions that target arousal systems. In fact, as discussed below, there is extensive evidence in both humans and rodents that odors presented during sleep can modulate memory consolidation, strongly suggesting that while some aspects of odor processing are gated by sleep, other aspects continue.

## 4 Sleep and memory across systems

### 4.1 Hippocampal and thalamocortical systems

Sleep plays an important role in memory in many systems where it has been examined (Walker and Stickgold, 2004; Diekelmann and Born, 2010), including visual discrimination (Gais et al., 2000), auditory memory (Antony et al., 2012), spatial memory (Rudoy et al., 2009), vocabulary learning (Koroma et al., 2022), paired associate memory (Marshall et al., 2004), and motor skill learning (Walker et al., 2002; Eckert et al., 2020; Elmaleh et al., 2021). For example, hippocampal dependent memory, such as for spatial location or context, is consolidated during nREM sleep (Wilson and McNaughton, 1994). In rodents, this sleep-dependent consolidation involves spontaneous replay of neural sequences that occurred during initial learning of a maze for example, which strengthens connectivity between neurons in the ensemble that encoded that activity (Skaggs and McNaughton, 1996; Lee and Wilson, 2002). This hippocampal replay generally occurs during hippocampal sharpwaves (Roumis and Frank, 2015; Drieu and Zugaro, 2019), which can coincide with neocortical slow-waves during nREM sleep in humans and animal models (Schwindel and McNaughton, 2011; Drieu and Zugaro, 2019; Jiang et al., 2019). The temporal association between hippocampal activity and cortical up-states may allow transfer of information from the hippocampus to the neocortex for long-term storage or reinforcement of existing neocortical ensemble connections (Schwindel and McNaughton, 2011; Drieu and Zugaro, 2019).

In addition to this spontaneous replay, imposing replay (also called targeted reactivation) or presenting contextual cues present during learning can also facilitate sleep-dependent memory consolidation. For example, in one paradigm subjects learned to play two different simple melodies and then during post-training nREM sleep had one of the melodies replayed through targeted reactivation. After waking, memory for the replayed melody was significantly better than for the non-replayed melody (Antony et al., 2012). In a study of visual spatial memory, subjects had to learn the location of visual objects on a screen in a spatial learning task where each visual cue was also associated with a unique sound (e.g., an image of a cat was paired with a meow sound) (Rudoy et al., 2009; Batterink et al., 2016). The subjects were then allowed to take a nap and during nREM sleep the subjects received targeted replay of a subset of the auditory cues. Subsequently, the subjects showed better spatial memory for visual stimuli whose auditory cues were replayed during sleep.

This form of targeted memory reactivation differs from more general manipulations of sleep that can also improve memory consolidation. Simply providing an opportunity for sleep between training and testing can improve memory (Walker and Stickgold, 2004; Wamsley et al., 2010). In addition, using transcranial stimulation to enhance slow-wave amplitude and sleep spindle power during nREM sleep can enhance hippocampal-dependent memory for paired associate word lists (Marshall et al., 2004, 2006) perhaps through facilitation of spontaneous replay.

Along with other factors, age seems to also play an interesting role in the interplay between odor memory and sleep. Prehn-Kristensen et al. (2015) compared the performance of children and adults in an incidental odor recognition paradigm, where half of the adults and half of the children went through the training phase in the evening and the testing phase in the morning, sleeping in between the two, while the other half of the two groups trained in the morning and was tested in the evening, without sleeping in between the sessions. Only the adult group showed significantly improved performance at recognizing odors after sleeping than without sleeping after the training phase; instead, the children’s performance was similar to the non-sleep adults group regardless of the presence or absence of sleep in between the two sessions. In another study, this time on older adults, sleep quality measured *via* both actigraphy and self-reported measures was associated to the ability to perceive and identify odors: the authors reported a decline in the odor identification (but not in the sensitivity to odors) in participants who showed lower sleep quality (McSorley et al., 2017). Therefore, the ability of odors and sleep to affect memory might not be evident in the early stages of life, but it might actually be relevant for adults and older adults, with the potential to associate odor training and memory consolidation with other therapies to prevent rapid cognitive decline.

Together this work suggests that memory is enhanced by sleep, improving sleep quality can improve memory consolidation in general, and targeted reactivation of memories during sleep can enhance specific memories. It also suggests that despite sleep related sensory gating, sensory input during sleep is sufficiently robust to affect ongoing activity and plasticity in diverse systems. However, all of these paradigms focused on hippocampal and/or neocortically based sensory modalities and memories. Is olfactory associative and perceptual memory, which involves circuits distinct from those just described, also modulated during sleep?

### 4.2 Olfactory system

Two primary approaches have been utilized to explore the interaction between odors and sleep-dependent memory that parallel those used in other systems. The first examines whether consolidation of memory of odors is sleep-dependent involving replay during nREM sleep, and the second examines whether targeted reactivation of odor cues presented during sleep can modulate memory consolidation. Sleep appears involved in odor memory consolidation given that the strength of odor memory is positively correlated with the amount of time spent in nREM sleep after odor learning (Barnes et al., 2011).

Work in both humans and animal models demonstrates that memory for odors and its underlying neural plasticity can be modulated during sleep (Barnes and Wilson, 2014a; Shanahan and Gottfried, 2014; Yamaguchi, 2017). In rats for example, there is evidence consistent with spontaneous replay of odor-induced activity within piriform cortical ensembles during nREM sleep. Similar to the hippocampus, the piriform cortex displays sharp-wave activity during nREM sleep and in some anesthetized states (Murakami et al., 2005; Wilson, 2010; Manabe et al., 2011; Katori et al., 2018). These piriform cortical sharp-waves are coincident with up-states in neocortical slow waves recorded in orbitofrontal cortex (Onisawa et al., 2017), as well as driving strong top-down input to the olfactory bulb and tubercle (Narikiyo et al., 2014). Thus, piriform cortical sharp-waves could facilitate information transfer and/or reinforce ensemble connectivity involved in memory consolidation, similar to that observed in hippocampal-dependent memory.

During nREM sleep when the piriform cortex appears less responsive to odors, piriform neurons fire in phase with sharp-waves with different neurons reliably active at different phases of the wave (Wilson, 2010; Katori et al., 2018). Importantly, odor learning/exposure during fast-wave (non-sleep) state can shift the timing of activity during the sharp-wave of single-units that were responsive to that odor (Wilson, 2010). It has been hypothesized that these timing shifts during sleep could allow repeated temporal association of activity in cells that were co-activated by an odor during waking, enhancing ensemble representation of that odor (Wilson, 2010). Thus, odor learning can modify piriform cell activity during subsequent slow wave sleep. Is this experience-dependent change in activity during sleep a form of spontaneous odor replay?

To test the sleep-dependent odor replay hypothesis in rodents, animals were trained to discriminate between two different spatial patterns of olfactory bulb activation (“electric odors”) in a fear conditioning task (Barnes and Wilson, 2014b). This direct electrical stimulation of the olfactory bulb neurons instead of natural odor-evoked neural activity allowed consistent re-activation of the olfactory system across vigilance states. Following training, animals were allowed to sleep, with some animals receiving additional CS + electric patterned olfactory bulb neural stimulation (i.e., imposed replay/targeted reactivation) during post-training waking, some during nREM sleep, and some receiving no additional stimulation. Targeted reactivation during post-training nREM sleep enhanced memory the following day. In contrast, the identical replay imposed during post-training waking induced memory extinction. Interestingly, introducing activity of a novel pattern of electrical olfactory bulb stimulation during post-training nREM induced fear of a stimulus the animals had never experienced before. Together, these results are consistent with a role for replay during nREM sleep in the strengthening of olfactory memory (Barnes and Wilson, 2014a).

Such sleep-dependent activity can affect plasticity within the piriform cortex itself, as well as targets of piriform output. For example, association fibers within the piriform cortex are highly plastic and believed to be important in odor coding and memory (Haberly, 2001; Poo and Isaacson, 2011). Pharmacological blockade of piriform association fiber connectivity during post-training sleep impairs the accuracy of subsequent odor memory (Barnes and Wilson, 2014b). The olfactory bulb is also modified by odor exposure and learning, and strong top-down input to the olfactory bulb from the piriform is involved in that plasticity (Yamaguchi et al., 2013; Yamada et al., 2017). For example, after receiving a meal during food restriction, adult generated olfactory bulb granule cells show increased apoptosis (Yokoyama et al., 2011). This increase in cell loss may be related to refinement in olfactory bulb coding of food odor. The cell loss is most extreme during post-feeding nREM sleep, and is hypothesized to be driven by odor-specific top-down input during piriform cortex sharp-waves during nREM sleep (Yamaguchi et al., 2013). The impact of piriform generated sharp-wave input to other areas during the sleep-dependent memory consolidation period, such as the orbitofrontal cortex (Onisawa et al., 2017), are unknown. This sleep-dependent pruning of neurons and connections in a circuit with lifelong neurogenesis is reminiscent of the hypothesized role for sleep in pruning and refinement of other circuits during early development, and in maintaining synaptic homeostasis in adults (Tononi and Cirelli, 2014).

Together, these rodent results suggest an important role for post-learning sleep in odor memory and memory-related plasticity in both the olfactory bulb and olfactory cortex, with evidence consistent with odor-replay during nREM sleep. Similar effects have been observed in humans. Similar to the animal model data, Arzi et al. (2012) found that humans could learn about odors presented during sleep. Pleasant or unpleasant odors associated with different frequency tones were presented to sleeping subjects. During waking, pleasant odors evoke larger inhalations than unpleasant odors (Bensafi et al., 2003), and tones associated with those pleasant or unpleasant odors can modulate respiration in a similar manner. Tones associated with pleasant odors during sleep (REM or nREM) evoked large sniff volumes both during sleep and during subsequent waking (Arzi et al., 2012), suggesting that the association between an odor and tone could be learned during sleep, modifying subsequent respiratory responses to the tone.

In contrast to learning new odor associations during sleep, there is also evidence of extinction of previously learned odor associations induced by odor exposure during sleep in humans (Hauner et al., 2013). Subjects were trained in an odor contextual fear conditioning task and then during post-training slow wave sleep were exposed to the contextual odor. The longer the contextual odor was experienced during sleep the more the autonomic response to the fearful CS + was reduced (i.e., extinction). In contrast, unpaired exposure to the contextual odor during waking enhanced CS + induced autonomic responses (Hauner et al., 2013). These results differ substantially from the rodent work described above and from other odor contextual learning effects described below. Nonetheless, they align with the general observations that odor information can be processed during sleep, and that learning about odors can occur during sleep (Perl et al., 2017).

In addition to memory for odors themselves, the effects of contextual odors on declarative memory have also been extensively examined. For example, human subjects were trained on a declarative memory task the context of a background odor. Presentation of that odor while the subjects were in nREM sleep after training significantly improved memory for the task (Rasch et al., 2007). Similar odor presentations during REM sleep did not improve declarative memory (Rasch et al., 2007; Cordi et al., 2014), nor did presentation of a different odor (Rihm et al., 2014). Given that declarative memory is typically hippocampal-dependent, it was also demonstrated that these odor cues activate the hippocampus, even during nREM sleep (Rasch et al., 2007). In a more targeted memory reactivation test, Bar et al. (2020), used a word-spatial location paired associate task, with the visual cues presented selectively to one visual hemi-field or the other. This allowed unilateral stimulus presentation. In addition, contextual odor cues were also presented unilaterally to one nostril or the other, which allows at least some lateralization of the context. Unilateral odor cues were then presented during nREM sleep in a protocol that allowed limiting the odor cue to the same hemisphere as the visual information, while no odor cue was presented to the contralateral hemisphere effectively allowing no reactivation of information presented to that side. The results suggest that targeting memory for visual information stored in the hemisphere receiving reactivation odor cues was significantly enhanced compared to information in the un-cued hemisphere (Bar et al., 2020).

In summary, similar to thalamocortical systems, (1) sleep improves odor memory, (2) odor information is processed during sleep, (3) both the strength and specificity of odor coding and memory can be enhanced by sleep-dependent replay and/or reactivation, and (4) odors can be used to reactivate and modify non-olfactory declarative memories during nREM sleep. Thus, odors can modulate neural activity crucial for plasticity and memory even during sleep, despite their relatively poor ability to induce waking compared to thalamocortical sensory systems.

During waking, odors can also modulate emotion and arousal (Herz, 2009; Ballanger et al., 2019; Kontaris et al., 2020; Boesveldt and Parma, 2021) and the olfactory pathway, as outlined above, has close anatomical ties to many structures related to sleep-wake cycling. This suggests that odor could not only modulate processing that occurs during sleep, but also could modulate sleep itself. In fact, the clear evidence of odor processing during sleep in the context of odor memory suggests that olfactory stimulation could not only modulate the probability of sleep onset, but also sleep maintenance. This reciprocal relationship between sleep modulation of odor processing and memory, and odor modulation of sleep is examined in the next section.

## 5 Odor modulation of sleep

Sleep onset and maintenance can be influenced by sensory input. For example, ambient light levels can promote or impair sleep onset, in part through circadian rhythm entrainment (Phipps-Nelson et al., 2003; LeGates et al., 2014; Santhi and Ball, 2020). Similarly, pain can delay sleep onset (Taylor et al., 2007; Eban-Rothschild et al., 2017) and auditory stimulation (e.g., music) can reduce stress and pain (Bernatzky et al., 2011; Hauck et al., 2013; Feneberg et al., 2021), potentially decreasing latency to sleep, while auditory noise can impair sleep (Smith et al., 2022). Furthermore, during nREM sleep auditory stimulation (pink noise–broad spectrum noise with matched intensity across octaves) presented in phase with 1 Hz slow cortical oscillations enhances slow-wave amplitude, indicative of improved sleep quality (Ngo et al., 2013). In fact, sleep-dependent memory consolidation is enhanced by this sensory stimulation protocol (Ngo et al., 2013), though the effect is reduced with aging (Schneider et al., 2020).

Olfactory cues may also modulate sleep and arousal in humans and animal models. Odors can modulate basic physiology such as autonomic tone (Vernet-Maury et al., 1999; Kiecolt-Glaser et al., 2008; Nagai et al., 2014), thus an olfactory effect on sleep and arousal may not be surprising. Although this literature is relatively sparse, and in some cases relies on self-report, there is evidence of the efficacy of some odors to modify latency to sleep onset, sleep duration and quality, and/or latency to waken. There are more studies that have examined the ability of odors to reduce stress and induce relaxation or enhance arousal which may be expected to modulate sleep (Kontaris et al., 2020), though sleep has not always been directly assayed.

As a specific example of odor modulation of sleep, cedar oil odor presented over 48 h enhanced the duration of EEG-monitored nREM sleep and decreased waking time in rats compared to no odor, and in a shorter protocol decreased latency to slow-wave sleep onset in napping humans (Sano et al., 1998). Similarly, lavender oil presented prior to bed was associated with enhanced time spent in nREM sleep in humans compared to no odor, and specifically in females increased the latency to wake after first falling asleep (Goel et al., 2005). In a well-controlled study using polysomnography, several distinct odors (e.g., lavender or vanilla) presented during sleep increased the power of both delta oscillations and sleep spindles recorded during nREM sleep, indicative of improved sleep quality (Perl et al., 2016). In these studies odors were presented for extended times prior to or during sleep. However, shorter odor exposure may also affect sleep. In a pilot feasibility test of a smartphone-based olfactometer that can release scent after the phone detected sleep onset based on movement, subjects reported a significant subjective increase in sleep quality in the odor condition compared to no-scent condition (Amores et al., 2022).

More biologically meaningful odors can also modulate sleep. For example, maternal odor can soothe human newborn infants, reduce crying and induced feeding behaviors (Sullivan and Toubas, 1998), a suite of effects that can accelerate sleep onset. In contrast in rats, food odor can induce waking, and is more effective at inducing waking from slow-wave sleep if the rats are food deprived than if sated (Gervais and Pager, 1979, 1982). Waking can also be induced by aversive odors in some subjects. Individuals with high chemical olfactory and/or trigeminal sensitivity/intolerance show reduced sleep duration and a trend toward longer sleep onset latency assessed with polysomnography (Bell et al., 1996).

Together, these findings suggest that odors can modulate sleep latency, quality and duration. What are potential mechanisms of this modulation?

### 5.1 Potential mechanisms of olfactory modulation of sleep

At a neural circuit level, there are multiple interconnections between primary olfactory structures and sleep networks which could provide direct or indirect odor-evoked modulation of sleep-wake cycling or stability. Most of the known connections are with wake promoting circuits. For example, odors, especially those that have been associated with reward, can activate both the noradrenergic locus coeruleus (Bouret and Sara, 2005) and the serotonergic dorsal raphe nucleus (Liu et al., 2014), two components of the wake promoting circuit that can induce cortical high frequency activity (Pace-Schott and Hobson, 2002; Saper et al., 2010). Similarly, activity of specific neural populations of the basal forebrain cholinergic system modulate odor processing and perception (de Almeida et al., 2013). However, those same cholinergic neurons can be driven by inputs from the olfactory system (Linster and Hasselmo, 2000), potentially allowing odor-evoked enhancement in arousal and wakefulness. In addition, the dopaminergic ventral tegmental area (VTA) which enhances wakefulness when activated and reduces wakefulness when suppressed (Eban-Rothschild et al., 2016), receives a strong olfactory input from the olfactory tubercle (Wesson and Wilson, 2011).

Though these studies have focused on odor-evoked excitation, odor-evoked suppression of any of these systems could potentially reduce arousal and wakefulness. In fact, in *Drosophila*, activation of the specific neurons within the olfactory pathway can regulate sleep in a bidirectional manner (Hsu et al., 2021). Similarly in mammals, orexin can regulate sleep and waking, with enhanced orexin activity promoting waking and decreased orexin activity promoting sleep (Ohno and Sakurai, 2008; Coleman et al., 2017). In mice orexinergic circuit activity can be regulated by olfactory stimuli such as linalool odor (Tashiro et al., 2016; Higa et al., 2021) and in turn the olfactory system is modulated by orexinergic input (Julliard et al., 2017). Other potential targets of olfactory output to sleep/wake circuits include the ventrolateral pre-optic area, a component of the sleep-wake switch circuit (Saper et al., 2010), which may receive a weak, indirect, olfactory input *via* GNRH neurons that are targeted by input from piriform cortex (Boehm et al., 2005). Whether such effects could be induced in humans at tolerated odor concentrations is unknown.

Furthermore, even in the absence of direct interaction between the olfactory system and sleep promoting circuits, reductions in pain perception, stress, or anxiety and/or increasing calmness could increase the likelihood of sleep onset. A number of odors have been shown to have anxiolytic or analgesic properties (Herz, 2016; Ballanger et al., 2019), and some odors can reduce physiological stress responses (Lee et al., 2022; Mori and Sakano, 2022). Co-occurrence of odor-induced anxiolysis and sleep promotion have been reported, though causal relationships have not been determined (Cho et al., 2013; Lytle et al., 2014; Karadag et al., 2017; Weston-Green et al., 2021). Such effects would not necessarily be aromatherapy in nature, i.e., dependent on a specific biomolecular effect, but an associative effect of personal odor preferences and responses.

Another feature of sleep that has received some attention is the way odors can modulate dreams. However, the results are quite inconsistent. In some cases, there was no detected link between odor presentation during sleep and dream content. For example, Martinec Novakova et al. (2021) found that neither pleasant (vanillin) nor unpleasant (thioglycolic acid) odors, compared to an odorless control, presented in both REM and nREM sleep stages, were able to affect the content of their participants’ dreams, measured *via* self-reported statements. When odors do affect dream content, it is usually in terms of their valence rather than direct incorporation of the odor in the dream, and even in this case, results fail to be consistent. For example, pleasant (phenyl ethyl alcohol) and unpleasant (hydrogen sulfide) odors (respectively, of roses and rotten eggs) produced significantly more or less pleasant dreams compared to a non-odor condition, respectively, when the odors were presented during the REM stage of sleep (Schredl et al., 2009). However, Okabe et al. (2018) also delivered phenyl ethyl alcohol in two different subgroups of participants (half liked the smell of roses, half did not) during the REM stage of sleep, and found that the participants who liked the smell reported more negative dreams compared to a control group who smelled an odorless control. Possible explanations for the low ability of odors to affect dreams might lie in (1) the limited efficacy of odor processing during REM sleep (Carskadon and Herz, 2004), (2) the impaired ability that humans have to describe odors: while we are good at describing other sensorial experiences (like visual or auditory stimuli), it is generally harder to describe smells, hence it is even harder to replay or recreate them in a dream (Nir and Tononi, 2010) or imagine them in waking (Stevenson and Case, 2005).

A final indirect relationship between olfaction and sleep in related to the ability of odors to modulate respiration. Respiration rate varies depending on O_2_/CO_2_ levels to enhance O_2_ availability during behavioral activity. Respiration rate can also affect cortical and behavioral activity in non-olfactory regions (Zelano et al., 2016; Heck et al., 2019; Girin et al., 2021; Tort et al., 2021; Juventin et al., 2022). Neurons in the pre-Botzinger’s complex of the brainstem help regulate breathing rate and a subset of these neurons project to the noradrenergic locus coeruleus which projects broadly through the brain. During rapid breathing, pre-Botzinger’s complex input to the locus coeruleus promotes cortical arousal, high frequency, low amplitude EEG, and behavioral arousal. In contrast, slow breathing reduces locus coeruleus excitation and promotes large amplitude, low frequency cortical oscillations commonly associated with calmness (Yackle et al., 2017). Thus, the forebrain has access to information about respiration rate *via* both the mechanosensory sensitivity of olfactory sensory neurons’ modulation of the olfactory system (Grosmaitre et al., 2007) and brainstem respiratory neuron modulation of the locus coeruleus (Yackle et al., 2017). Odors can also modulate respiration in patients with brain injuries that are otherwise unresponsive (Arzi et al., 2020). In fact patients that show odor-induced respiratory responses have a much better prognosis for recovery than those that do not (Arzi et al., 2020). Given the ability of odors to modulate respiration (Johnson et al., 2003; Masaoka et al., 2005; Arzi et al., 2010), odors that reduce respiratory rate could reduce cortical arousal and thus, indirectly contribute to the reported odor-induced enhancement of sleep onset and/or maintenance (Masaoka et al., 2005; Perl et al., 2016).

## 6 Summary, remaining issues, and future directions

**FIGURE 3:**
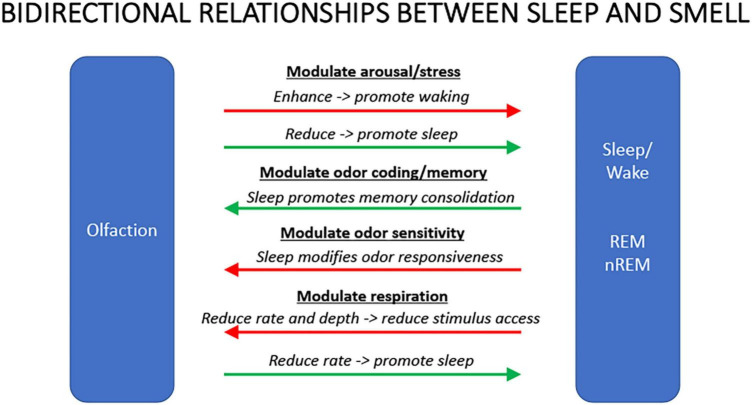
Summary schematic of some of the major bidirectional interactions between olfaction and sleep. Odors that can enhance arousal or stress will promote waking and/or delay sleep, duration, or quality. Conversely, odors that reduce arousal or stress will promote sleep. Sleep, in turn, can facilitate odor memory consolidation though it can reduce olfactory system sensitivity to ongoing odors. Finally, odors that reduce respiration rate can reduce activity in circuits promoting wakefulness leading to increased probability of sleep. During sleep, respiration rate and depth can impair odor access to sensory neurons contributing to reduce olfactory system responsiveness.

As in other sensory systems, despite major anatomical differences, sleep plays important roles in olfactory function including modulation of odor sensitivity and serving as an important regulator of both perceptual and associative olfactory memory storage. However, olfaction also has an important modulatory role over sleep. Odors can modulate the latency to sleep onset, as well as the quality and duration of sleep. Olfactory modulation of sleep may be mediated by direct synaptic interaction between the olfactory system and sleep control nuclei, and/or indirectly through odor modulation of arousal and respiration. Such modulation appears most heavily influenced by past associations and expectations about the odor, beyond any potential direct physicochemical effect. Nonetheless, the reciprocal interaction between sleep and olfaction (Figure 3) presents novel opportunities for sleep related modulation of memory, as well as development of non-pharmacological olfactory treatments of simple sleep disorders.

However, there are many unresolved questions regarding smell and sleep interactions. For example, while it is clear that general behavioral responsiveness to some odors is reduced during sleep, odor stimulation during sleep can still activate central structures such as the hippocampus, and can modulate some aspects of memory consolidation. Thus, any sleep-dependent olfactory sensory gate is porous, as is increasingly observed in thalamocortical sensory systems. Given the diverse targets of olfactory bulb outputs, and evidence for distinct olfactory bulb cell populations providing parallel outputs to these targets, are there differences in state-dependent modulation in these pathways? Are some olfactory pathways more susceptible to sleep-dependent sensory gating than others? What are the relative contributions of peripheral and central state-dependent changes in olfactory information flow?

An additional important note is that the vast majority of the research on odor and sleep has focused on the use of single ingredients. These ingredients range from floral smells like lavender (Goel and Lao, 2006; Jung and Choi, 2012) to herbal smells like chamomile (Srivastava et al., 2010). Other odor families such as menthols (e.g., peppermint, see Norrish and Dwyer, 2005) can, on the contrary, reduce sleepiness and promote wake, although these results have been inconsistent, and might actually depend on odor preference and other parameters like gender (Goel and Lao, 2006). Similar results have been obtained with mixtures of two or three ingredients (for a review, see Hwang and Shin, 2015). Other studies employed more complex, albeit “personalized” smells to determine the influence of odors on sleep. Hofer and Chen, for example, reported increased perceived (self-report) and measured (actigraphy) sleep quality in participants sleeping with a t-shirt worn by their partner compared to a control (either a clean t-shirt or one worn by a stranger) (Hofer and Chen, 2020). However, there is a gap in the current literature with regard to fully formulated fragrances, as the landscape is dominated by studies employing single ingredients or blends of very few ingredients.

Future work on odor modulation of sleep onset and quality must include high quality research designs including randomized controls of the odors used and control odors. Very few existing studies of odor modulation of sleep include control odors, but rather are comparisons between odor presentation and no odor, which provides no insight into which features of odor stimuli are most effective at sleep promotion. What are the relative contributions of odor physicochemical qualities and subject expectations on the efficacy of odors to modulate sleep? Furthermore, use of polysomnography to help identify potential variation in odor affects across sleep stages would be useful, again to help identify mechanisms of odor effects. Clinical studies of odor effectiveness across different sleep disorders and across healthy aging should also be targeted for future work. Finally, where possible, mechanistic studies should target not only animals models but also diverse human subjects across the lifespan.

## Author contributions

Both authors contributed to the writing of this review manuscript and figure preparation, contributed to the article, and approved the submitted version.
